# Assessment of aneuploidy formation in human blastocysts resulting from cryopreserved donor eggs

**DOI:** 10.1186/s13039-015-0117-8

**Published:** 2015-02-15

**Authors:** Aimin Deng, Wei-Hua Wang

**Affiliations:** Center for Reproductive Medicine, Changsha Hospital for Maternal and Child Health Care, No. 416, Chengnan East Road, Changsha City, Hunan China; Houston Fertility Laboratory, Vivere Health, Houston, TX USA; Houston Fertility Institute, 2500 Fondren Road, Suite 350, Houston, TX 77063 USA

**Keywords:** Aneuploidy, Egg freezing, Donor eggs, Implantation

## Abstract

**Background:**

Increased embryo implantation rates were reported after transfer of euploid embryos selected by preimplantation genetic screening (PGS). Egg cryopreservation by vitrification has become one of the most important assisted human reproduction technologies. Although reports indicate that development and implantation of human embryos derived from frozen donor eggs are comparative to fresh eggs, it is still unknown whether egg vitrification increases chromosomal abnormalities in eggs, which in turn causes formation of embryonic aneuploidy. Therefore, in this study, we evaluated the aneuploidy formation in the blastocysts derived from frozen donor eggs and also evaluated the efficiency of egg vitrification as an advanced technology for egg cryopreservation.

**Results:**

In this study, donated human eggs from young women were cryopreserved by vitrification and PGS was performed in the resulted blastocysts by DNA microarray. A total of 764 frozen eggs from 75 egg thawing cycles were warmed and 38 blastocysts were biopsied for PGS before embryo transfer. A 97.1% of egg survival rate was obtained and 59.1% of embryos developed to blastocyst stage. After biopsy and PGS, it was found that 84.2% of blastocysts were euploid and 15.8% were aneuploid. Aneuploidy rates varied among donors. Transfers of blastocysts without PGS resulted in higher clinical pregnancy and implantation rates as compared with transfer of blastocysts with PGS.

**Conclusions:**

Although the overall aneuploidy rate was low in the blastocysts derived from frozen donor eggs, high aneuploidy rates were observed in the embryos resulting from some donated eggs. Clinical pregnancy rate was not improved by PGS of embryos resulting from donor eggs, indicating that PGS may not be necessary for embryos derived from donor eggs in most cases.

**Electronic supplementary material:**

The online version of this article (doi:10.1186/s13039-015-0117-8) contains supplementary material, which is available to authorized users.

## Background

Egg cryopreservation has become one of the key assisted reproduction technologies (ART) in humans [1-7]. Successful egg cryopreservation has allowed for the establishment of frozen egg banks [8-10]. A few studies have reported high clinical pregnancy and live birth rates after transfer of the resulting embryos with vitrified/warmed human eggs [7-11]. It has also been reported that embryo development, clinical pregnancy and embryo implantation rates are similar between fresh and cryopreserved eggs in some egg donation programs [7,12-14]. There was no apparent increase in congenital anomalies [15] and other abnormities [4] in the babies derived from frozen eggs. Based on the accumulated clinical data published in recent years, the American Society for Reproduction Medicine has opined that oocyte cryopreservation should no longer be considered experimental since 2012.

Previously it has been found that freezing can cause spindle abnormalities [16] and chromosome misalignment [17] in human eggs, which in turn may increase the occurrence of aneuploid embryos after fertilization [18-21]. When vitrification, a new egg cryopreservation technology, was compared with slow egg freezing, it was found that vitrified eggs had a better survival rate than slow freezing [22]. The morphology, meiotic spindle, DNA integrity and gene expression in human eggs are also different between vitrification and slow freezing [23,24]. These data indicate that vitrification is more efficient to cryopreserve human eggs than slow freezing. Although Coticchio et al. reported that vitrification may also increase chromosome misalignment in human eggs [17], it was noticed that ultra-structure alternation in human eggs after vitrification was related to vitrification methods, especially cooling rate [25]. Recently, Forman et al. reported that vitrification did not increase embryonic aneuploidy formation in young women undergoing in vitro fertilization (IVF) [26]. This may indicate that human eggs can recover normally (structure and function) after minor changes caused by vitrification and warming if optimal methods are used.

Aneuploidy is one of the most essential factors affecting embryo implantation and most of the birth defects are also caused by embryonic aneuploidy [18,19]. It has been reported that aneuploidy rate in preimplantation embryos is increased in patients of advanced maternal age [19,21,27]. Recently, it has also been found that high proportions of human embryos were aneuploid in younger patients undergoing IVF [28,29]. Donor eggs are usually collected from healthy, young and fertile women and should have normal quality and chromosomal integrity. However, these eggs are collected after controlled ovarian stimulation with high dosages of external gondotropins and then the eggs are exposed to in vitro environments for manipulations; many aspects, especially the genetic and epigenetic alternations, are still unknown.

Preimplantation genetic screening (PGS) by all chromosome DNA microarray has become available in human ART, which has provided an approach to examine structural and numeral abnormalities in all chromosomes in the preimplantation embryos [27,29-31]. It has been found that PGS especially benefits the patients of advanced maternal ages, recurrent miscarriage and previous spontaneous miscarriage, as aneuploidy is the main reason for unsuccessful embryo implantation in these populations of patients [27,30-33]. However, such information is still missing in the patients who receive the cryopreseved donor eggs. If vitrification can cause some meiotic spindle and/or chromosomal alternations, PGS may be useful for selecting normal embryos. Therefore, in the present study, we retrospectively collected data on PGS of blastocysts derived from frozen donor eggs and aimed to analyze the aneuploidy in human embryos resulting from cryopreserved donor eggs and also to evaluate the efficiency of egg vitrification as an advanced technology for egg cryopreservation.

## Results

### Egg survival and embryo development

As shown in Table 1, a total of 647 eggs for 75 recipient cycles were warmed during this data collection period and each recipient received average of 8.6 ± 1.4 eggs (range from 5–11). After warming and insemination, 627 eggs (97.1%) survived and 535 (85.3%) fertilized normally (2 pronuclei). Out of 477 (89.2% cleavage rate) cleaved eggs, 282 developed to blastocysts with a blastocyst formation rate of 59.1%.

**Table 1:** **Summary of the outcome of donor egg cryopreservation/warming cycles**

**Total warming cycles**	**75**
Average age of recipients	41.6 ± 4.83^#^
Total No. of eggs warmed	647
Average No. of eggs per recipient	8.6 ± 1.4^#^
Total No. of eggs survived (%)*	627 (97.1)
Total No. of eggs fertilized (%)**	535 (85.3)
Total No. of eggs cleaved (%)***	477 (89.2)
Total No. of blastocysts (%)****	282 (59.1)

^#^Mean ± SD.

*Percentage of warmed eggs.

**Percentage of survived eggs.

***Percentage of fertilized eggs.

****Percentage of cleaved eggs.

### Aneuploidy in the blastocysts derived from frozen donor eggs

**Figure 1:**
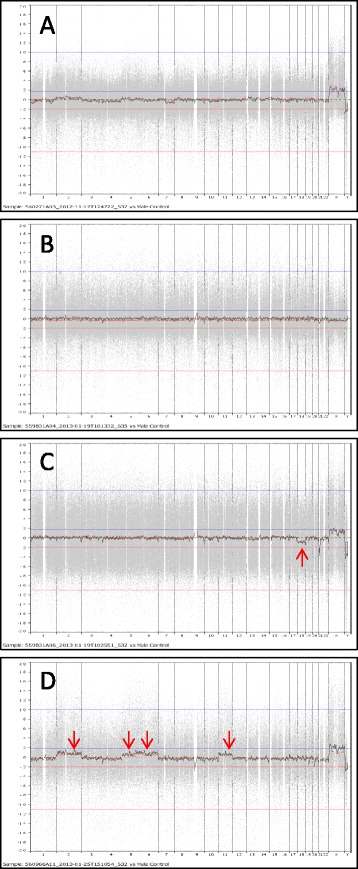
**Microarray charts of blastocysts derived from vitrified donor eggs. A)** arr(1–22,X) × 2 **B)** arr(1–22) × 2,(XY) × 1 **C)** arr(18) × 1; **D)** arr(2) × 3,(5) × 3,(6) × 3,(11) × 3. Red arrows indicate abnormal chromosomes.

As shown in Table 2, 8 recipients received 74 eggs from 8 anonymous donors. The donor ages were between 21–31, and 199 eggs (from 10–51 per donor) were retrieved and 179 (90.0%) were matured at Metaphase II stage. Eight recipients aged between 40 and 52 years old received 74 eggs that were warmed with a 97.3% survival rate. After insemination, 64 (88.9%) eggs fertilized normally, 56 (87.5%) cleaved and 38 (67.9%) developed to blastocysts. Blastocyst rates were 25-80%. All blastocysts were biopsied and DNA microarray was performed for aneuploidy screening. Out of 38 blastocysts, 32 (84.2%) were euploid, and 6 (15.8%) were aneuploid. No aneuploid embryo was observed in four cases, while 12-50% of aneuploid embryos were observed in other 4 cases. Some PGS charts were shown in Figure 1 in which euploid and aneuploid samples were presented.

**Table 2:** **DNA microarray analysis of all chromosomes in the blastocysts produced from vitrified donor eggs**

**Case #**	**1**	**2**	**3**	**4**	**5**	**6**	**7**	**8**	**Total (%)**
Donor age	29	22	21	24	23	29	22	31	25.1 ± 3.9*
Total gonadotropins	3450	3000	1725	1725	1725	1725	4275	2250	
E_2_ at trigging day	3426	4244	5228	3928	8398	4238	2485	9046	
Trigger	Ovi	Ovi	Lup	Lup	Lup	Ovi	Ovi	Lup	
No. of eggs retrieved	22	51	18	10	28	28	21	21	199
No. of eggs matured	20	45	14	10	27	28	15	20	179 (90.0)
Age of recipient	51	41	43	40	43	48	44	52	45.3 ± 4.5*
No. of eggs warmed	10	10	10	10	8	8	8	10	74
No. of eggs survived	10	8	10	10	8	8	8	10	72 (97.3)
No. of eggs fertilized	8	7	9	10	7	7	6	10	64 (88.9)
No. of eggs cleaved	8	5	4	10	7	6	6	10	56 (87.5)
No. of blastocysts	6	4	1	8	4	3	4	8	38
% of blastocyst	75	80	25	80	57	50	67	80	(67.9)
No. of euploid blasts	6	3	1	6	2	3	4	7	32
% of euploid blasts	100	75	100	75	50	100	100	88	(84.2)
No. of aneuploid blasts	0	1	0	2	2	0	0	1	6
% of aneuploid blasts	0	25	0	25	50	0	0	12	(15.8)

Ovi: Ovidrel; Lup: leuprolide acetate.

*Mean ± SD.

When we investigated whether stimulation had a correlation with aneuploid embryos from donor eggs, as shown in Table 2, we found that aneuploid embryos were present in 3 out of 4 donors who had leuprolide acetate trigger due to high level of E_2_ while aneuploid embryos were present only in 1 out of 4 donors who had regular hCG (Ovidrel) trigger. This is not related with the total dosage of gonadotropins used, but it may depend on the individual response (such as high estradiol level) to external gonadotropins. Detailed chromosome information of all abnormal embryos was listed in Table 3. Abnormal embryos were not reanalyzed due to cost reasons.

**Table 3:** **Detailed chromosome information of the abnormal embryos derived from donor eggs**

**Sample number**	Chromosome information*
1	arr(18) × 3
2	arr(8) × 1
3	arr(2) × 3, (5) × 3, (6) × 3, (11) × 3
4	arr(13) × 3
5	arr(3) × 1, (4) × 1, (14) × 1, (10) × 3, (11) × 3
6	arr(X) × 1

^*^Microarray chromosome information was named by referring to Cytogenetic Nomenclatures ISCN 2013 [44].

### Pregnancy after transfer of embryos derived from frozen donor eggs

In the present study, 63 cycles had embryo transfers without PGS, 43 (68.3% per transfer) became biochemically pregnant which was examined about two weeks after transfer; 40 (63.5% per transfer) became clinically pregnant and 36 (57.1% per transfer) delivered normal healthy babies. These data were based on the first fresh embryo transfer in these patients. Seven patients had embryo transfer after PGS with either fresh and/or frozen embryos, 4 (57.1% per transfer) transfers resulted in biochemical pregnancy and 3 became clinically pregnant (42.9% per transfer) and delivered normal healthy babies (42.9%). Four patients without PGS and one patient with PGS did not have embryo transfer so far, so there is no clinical data available.

## Discussion

One of the benefits of egg cryopreservation is the establishment of frozen donor egg banks in which recipients and egg donors do not need to synchronize the same cycle, and recipients can select their donors and/or eggs whenever they need. Another benefit is that many eggs are usually retrieved from young egg donors and it is not necessary to use all eggs to make embryos for one recipient, and supernumerary eggs can be frozen for other recipients to use. This makes egg sharing donation particularly easy among recipients [34,35].

Similar to previous studies [1-7], the present study indicates that high survival rates can be obtained after eggs were cryopreserved with vitrification. Also, high blastocyst development and embryo implantation rates were obtained with the vitrified/warmed donor eggs. In the present study, we also provide the first evidence that high proportions of blastocysts derived from frozen donor eggs were euploid after PGS. This may indicate that meiotic spindles recovered normally during vitrification and chromosomes segregate correctly after vitrification/warming. As of our knowledge, this is the first study to be performed to screen aneuploidy in the embryos derived from vitrified donor eggs.

Egg vitrification, as a new cryopreservation technology, has been used in human IVF for a few years and there are still many concerns, such as viability (not just survival rate), genetic stability and epigenetic status [24]. The present study indicates that meiotic spindles and/or chromosomes may be quite stable in the young donor eggs after vitrification and warming as shown by low aneuploidy rate in the derived blastocysts. Although such a conclusion is based on a limited number of embryos (38 blastocysts), it provides information that aneuploidy rate is very low in young fertile women (egg donors) and vitrification may not increase its rate. This conclusion is also supported by high implantation rates of embryos derived from donors [3,7,10-14], and from young fertile [35] and infertile women [26].

Aneuploid embryo formation is one of the major factors affecting human IVF success and such an affect could be reduced by transfer of euploid embryos [27,29]. An age-related reduced embryo implantation can also be overcome by the transfer of selected embryos with PGS [27]. Aneuploid embryos are present in infertile patients of various maternal ages with an increased tendency as maternal ages are increased [27,29,31,36]. Embryos from healthy egg donors should have low embryonic aneuploidy rates, but direct information is still missing due to the fact that high pregnancy rates can be obtained with donor eggs and PGS for embryos derived from donor eggs are not routinely performed due to cost and necessity.

By contrast, for egg cryopreservation, due to meiotic spindle’s sensitivity to temperature [37-39], it is possible that spindle damage occurs during egg in vitro manipulation [37-39] and cryopreservation [16,17]. Although vitrification can provide a better egg survival than slow freezing, the meiotic spindle and/or chromosomal distribution must have undergone dynamic changes due to temperature fluctuations during vitrification and warming. It is possible that suboptimal protocols may negatively influence the normal meiosis after egg cryopreservation [25]. It is necessary to functionally study meiotic spindle recovery and chromosome distribution in frozen eggs. However, due to the shortage of donor eggs for the related studies, such information is still unavailable. The alternative way to investigate the chromosomal changes after egg cryopreservation may be the examination of embryonic aneuploidy formation, which can indirectly provide information of whether the vitrification affects the meiotic spindle recovery, chromosome alignment, normal meiosis and subsequent mitosis without destroying eggs. Our present study with DNA microarray of blastocysts derived from frozen donor eggs indicates that aneuploidy rate is low in donor eggs and vitrification/warming may not increase the rate.

Other indirect evidence that vitrification/warming may not increase embryonic aneuploidy rates is the direct comparison of embryo implantation between fresh and frozen eggs from the same donors. Such studies have been performed in a few egg donor programs and no differences were observed in terms of blastocyst formation, pregnancy and implantation rates [3,7,10-13].

Recently, Forman et al. investigated the aneuploidy formation in human embryos derived from fresh and frozen eggs that were collected from the same young patients and found that vitrification did not increase aneuploidy formation either in arrested embryos or blastocysts [26]. This is the first direct study with DNA microarray to indicate that vitrification is a safe and effective procedure to preserve human eggs. In Forman’s study, the authors found that aneuploidy rate of human eggs after vitrification was about 20% [26], which is lower than that (40%) reported by Yang et al. [29]. Both studies examined the embryos from IVF patients. In the present study, we found that the aneuploid rate was 15.8% from the egg donors who are healthy young women. In the present studies, all embryos from 4 donors were normal euploid while 25-50% of embryos from the other four donors were aneuploid. The reasons for the aneuploidy in these eggs are still unknown but it may be related to donors or donor stimulation as there were variations among donors.

In a previous study, it was reported that extra ovarian stimulation can increase aneuploid formation in human IVF [40]. In the present study, we also found such a tendency that high estradiol level may be related to aneuploidy formation. However, a comprehensive conclusion may need more clinical data. These data may indicate that aneuploidy is a donor specific, not a procedure (vitrification) specific and that egg vitrification may not increase chromosomal abnormalities and current vitrification protocols for eggs are efficiency.

Blastocyst transfer has been widely adapted in many IVF clinics and it has also been suggested that blastocyst transfer is an option for egg thawing cycles [41]. Many IVF clinics still use Day 3 embryo transfer for egg thawing cycles, and this may be due to the worries of low blastocyst development with frozen eggs. However, based on a few direct comparison studies, it would appear that the same blastocyst development rates could be obtained with fresh and frozen human donor eggs [7,12-14].

In the present study, we did not find any benefit of PGS on the embryo implantation and/or baby delivery rates, while a lower birth rate was found if PGS was applied to the embryos from frozen eggs. There are a few possibilities to explain this situation. First of all, embryo biopsy may affect the embryo viability although such a procedure does not affect the viability of blastocysts derived from fresh eggs [27,29,31]. We found that slow embryo development was present in the vitrified eggs in some cases and the blastocysts did not expand completely at day 5, thus trophectoderm cell biopsy may affect the developmental potential of embryos in the early blastocysts. The second reason may be due to the delayed embryo transfer after biopsy and PGS. In the present study, we performed biopsy at day 5 and transferred the embryos at day 6 after the PGS results became available in some cases. The blastocyst viability may be affected by the procedures. However, this situation was not observed in Forman’s study [26], in which PGS was performed on site and the time for PGS on site can be reduced or biopsy may be performed only on expanded blastocysts, which is the optimal embryo stage to perform a biopsy.

## Conclusions

In conclusion, our study indicates that high egg survival rates and blastocyst development rates can be obtained with vitrified human donor eggs. Acceptable pregnancy and implantation can also be obtained with transfer of the blastocysts resulting from frozen donor eggs. Because donor eggs are from healthy and young women, the embryonic aneuploidy rate is low in this population of embryos and vitrification may not increase chromosomal abnormalities, aneuploidy screening may be not necessary in most cases. However, if the donors are over stimulated by external gonadotropins and have extremely high E_2_ levels, there may be an increased possibility to have some aneuploid embryos, and PGS may be useful to screen embryos. Further clinical studies remain necessary.

## Methods

### Ethics

Patients undergoing IVF, egg donation and PGS signed written consents for all kinds of laboratory and clinical procedures. All egg donors were anonymous in the present study. The data was retrospectively collected from the medical records and the study was approved by Institutional Review Board (NEIRB 14–504).

### Donor stimulation and egg retrieval

Egg donors were stimulated with a combination of Follistim (Organo Inc, Roseland NJ, USA), Gonal-F (EMD Serono, Rockland MA, USA), Menopur (Ferring Pharmaceuticals, Parsippany NJ, USA) and/or Bravella (Ferring Pharmaceuticals) beginning 2–3 days after the onset of menses. The initial starting total dose was 150–375 IU and was adjusted subsequently as the stimulation progressed. To prevent an luteal hormone surge, a GnRH antagonist, Ganirelix or Cetrorelix (Organo Inc.), was given when the leading follicle was 13–14 mm or when the estradiol level was 400 pg/ml. Human chorionic gonadotropin (hCG), Ovidrel (Serono USA), or a GnRH agonist, leuprolide acetate (Teva North America, North Wales PA, USA), was injected to induce final oocyte maturation when at least two dominant follicles reached a diameter of >18 mm. Eggs were retrieved under IV sedation via transvaginal ultrasound between 35–37 hours after hCG or leuprolide acetate administration. Supernumerary oocytes were cryopreserved and stored in an egg bank if they were not used freshly for insemination for the recipients who were prepared for embryo transfer. In some cases of donation, all eggs were cryopreserved and placed into the egg bank.

### Egg vitrification

Oocytes were cultured for 3–5 hours before removing the surrounding cumulus cells in a HEPES-buffered global medium (IVFonline, CT, USA) containing 40 iu hyaluronidase. Only mature (metaphase II) oocytes were vitrified with Irvine vitrification kits (Irvine Scientific, Irvine CA USA). All procedures were performed at room temperature (22-25°C) based on the procedures reported previously [7].

### Egg warming and insemination

Egg warming was based on the procedures previously reported [7]. Briefly, straws were removed from liquid nitrogen and the tips of the straws with eggs were quickly placed in 1 ml 1.0 M sucrose solution that had been warmed at 37°C in an organ culture dish. After 1 minute in the sucrose solution, eggs were transferred to 1 ml 0.5 M sucrose solution for 3 minutes and then to 1 ml basic solution for 2 × 5 minutes. These procedures were performed at room temperature (22-25°C). After warming, eggs were washed with Global medium supplemented with 10% serum protein substitute (SPS) (IVFonline) and then cultured in the same medium. Egg survival status was evaluated based on morphology after completion of the warming procedures. All survived eggs were inseminated by intracytoplasmic sperm injection (ICSI) 2–3 hours after warming.

### Embryo culture and embryo biopsy

All eggs were cultured in Global medium supplemented with 10% SPS after ICSI. Fertilization was examined 16–18 hours after ICSI and normally fertilized eggs (zygotes) were cultured in Global medium supplemented with 10% SPS at 37°C in a humidified atmosphere of 5.5% CO_2_, 5% O_2_ and balanced nitrogen until day 6 after inseminations. On day 5, embryo development was evaluated and the best 1–2 embryos depending on embryo quality were transferred. If the recipients were not ready for transfer or PGS was requested, day 6 embryo transfer (PGS cases) or frozen embryo transfer (FET) were performed.

For PGS embryos, at day 3, a hole about 20 μm in diameter was opened in the zona pellucida using the ZILOS-tk™ laser system (Hamilton Thorn Bioscience Inc., MA USA). On day 5, embryos were examined with an inverted microscope, and if trophectoderm (TE) cells started to hatch from the opening in the zona pellucida, some hatched TE cells (~10) were biopsied using a 20 μm polished biopsy pipette with assisted cutting by laser. Blastocyst biopsy was performed on TE cells at days 5 or 6 depending on blastocyst development. After biopsy, the embryo proper was cultured in Global medium supplemented with 10% SPS for 1–2 hrs before vitrification or continued in the culture until day 6 if a fresh embryo transfer was required. The biopsied cells were washed with a washing buffer provided by PGS laboratory (PacGenomics Inc, CA, USA), placed in tubes with cell lysis buffer and were then frozen at −20°C before being processed for microarray.

### Blastocyst vitrification, warming and embryo transfer

Blastocysts were vitrified after the blastocoele was completely collapsed according to a previous method [42] by using Irvine vitrification kit. Briefly, blastocysts were equilibrated in the equilibration solution for 2 minutes on a warming stage (37°C). The blastocysts were then transferred into the vitrification solution and then loaded onto a vitrification straw within 45 seconds. All embryos were vitrified individually and then stored in liquid nitrogen until warming for FET.

For warming, blastocysts were exposed to 1 M warmed (37°C) sucrose solution for 1 minute. Blastocysts were then transferred to 0.5 M sucrose solution for 3 minutes and to a basic solution for 10 minutes with a solution change after 5 minutes at room temperature. After completion of the warming process, blastocysts were washed with Global medium supplemented with 10% SPS and then cultured in the same medium for 2–4 hrs before transfer. Blastocyst quality was assessed using standard assessments developed by the Society of Assisted Reproductive Technology [43].

### Microarray of biopsied samples with oligo NimbleGen platform

Microarray of biopsied samples was performed based on a previous reported procedure [31]. Briefly, samples were lysed and the cells’ genomic DNA was amplified using Rubicon whole-genome amplification kit (Rubicon, MI, USA). The DNA concentration of purified samples was measured using Nanodrop 2000 (Thermo, DE, USA) and the samples were then labeled with Cy3 using the NG dual color labeling kit according to manufacturer’s instructions (Roche NimbleGen, IN, USA). Labeled samples were mixed with Cy5 control labeled samples, dried, dissolved and loaded on NimbleGen 6 × 630 K comparative genome hybridization (CGH) tiling array following the NimbleGen hybridization protocol. Reference DNA (both male and female were used) for array was obtained from Promega (Promega Corporation, Madison, WI USA). After overnight hybridization, arrays were washed following the NimbleGen washing protocol. Arrays were dried and scanned with a NimbleGen MS200 scanner (Roche NimbleGen, IN, USA) at 2 μM scanning resolution. Scanned images were analyzed by Deva 1.1 software (Roche NimbleGen, IN, USA) and the normalized ratio of each sample versus the control was retrieved following the NimbleGen CGH data analysis protocol. Finally, the normalized ratio of each sample was input into Nexus 6.1 software (Biodiscovery, CA, USA) and the Log2 ratio result of each sample’s whole genome view is presented. Human Genome Build 19 (hg19) was used in the present study. Microarray chromosome information was named by referring to Cytogenetic Nomenclatures ISCN 2013 [44].

### Patient preparation for embryo transfer

All patients for embryo transfer received estradiol orally and transvaginally. Intramuscular administration of progesterone oil was initiated after about 14 days of estradiol treatment. Endometrium thickness was measured on the day of progesterone administration. Embryo transfer occurred on the sixth or seventh day of progesterone administration and progesterone was continued until the first serum β-hCG test two weeks after transfer. Ongoing pregnancies were supported by continued estradiol and progesterone.

### Pregnancy and live birth rate assessment

Fourteen days after embryo transfer, pregnancy was checked by a serum β-hCG assay. When the β-hCG was > 5 mIU/mL the patients were regarded as having a biochemical pregnancy. Four weeks after embryo transfer, when a gestational sac and a heart beat appeared ultrasonographically, the patients were diagnosed as having a clinical pregnancy. The live birth rates indicated as live birth per embryo transfers.

## Abbreviations

ART: Assisted reproduction technology
IVF: In vitro fertilization
ICSI: Intracytoplasmic sperm injection
hCG: Human chorionic gonadotropin
GnRH: Gonadotropin release hormone
SPS: Serum protein substitute
TE: Trophectoderm
FET: Frozen embryo transfer
